# Whole-Genome Sequencing and Annotation of 10 Endophytic and Epiphytic Bacteria Isolated from Lolium arundinaceum

**DOI:** 10.1128/MRA.00317-21

**Published:** 2021-05-13

**Authors:** Victoria A. Gaeth, Christina J. Domondon, Paul A. Podbielski, Virginia X. Aswad, Emalee A. Wrightstone, Narayan H. Wong, Wilford H. Burke, Jo Melita, Kristen M. Murray, André O. Hudson

**Affiliations:** a The Thomas H. Gosnell School of Life Sciences, Rochester Institute of Technology, Rochester, New York, USA; University of Arizona

## Abstract

We report the whole-genome sequence and annotation of 10 endophytic and epiphytic bacteria isolated from the grass Lolium arundinaceum as part of a laboratory exercise in a Fundamentals of Plant Biochemistry and Pathology undergraduate course (BIOL403) at the Rochester Institute of Technology in Rochester, New York.

## ANNOUNCEMENT

Lolium arundinaceum, commonly known as tall fescue, is a primary turf grass located in temperate regions of the world (1). *L. arundinaceum* is an invasive species that is able to grow robustly under harsh conditions due to its adaptability to a variety of environmental niches (1, 2). This organism poses an ecological threat due to endophyte infections that likely confer a competitive advantage over other native plants (1). Approximately 75 to 90% of *L. arundinaceum* is infected with the symbiotic endophytic fungus Epichloё coenophiala (3, 4). This fungus provides stressor tolerance via various alkaloid compounds, some of which can lead to fescue toxicosis, that negatively affect grazing wildlife and livestock, resulting in reduced blood flow (vasoconstriction) and reproductive organ damage (3–5). Therefore, it is important to characterize the microbial content of tall fescue and other grasses to further understand the metabolites produced by endophytic organisms along with their impacts on the host and environment.

The microbiome of tall fescue has been previously characterized by methods including 16S rRNA gene sequencing and various enzymatic assays to identify their function as plant symbionts (6). Roles were discovered to include mineral solubilization, nitrogen fixation, and cellulase production (6). This study provides whole-genome sequencing and annotation of bacterial genomes to facilitate future studies related to plant-host interactions and their symbiotic relationships.

A 60-day-old sample of composted *Lolium arundinaceum* was used to inoculate broth medium, followed by 10-fold serial dilutions. The dilutions were plated on agar plates and incubated at 30°C for 48 h. Individual colonies were chosen based on size, color, and morphology to inoculate a 5-ml culture, followed by genomic DNA (gDNA) extraction using the GenElute bacterial genomic DNA isolation kit (Sigma-Aldrich, USA) according to the manufacturer’s protocol. Genomic DNA quality was assessed using a NanoDrop One device for *A*_260/280_ and *A*_260/230_ ratios. Samples of sufficient quality were quantified using a DNA high-sensitivity (HS) kit on a Qubit 3.0 device and diluted to a concentration of 0.2 ng/μl, and 5 μl of each sample was processed using an Illumina Nextera XT kit according to the manufacturer’s protocols. The average fragment length of each library was determined using a DNA HS kit on the Agilent 2100 bioanalyzer, and these data were combined with DNA concentration data from the Qubit 3.0 device to dilute libraries to 4 nM. Diluted libraries were pooled, denatured, and diluted to a concentration of 10 pM for loading onto the Illumina MiSeq instrument using an Illumina V3 600-cycle kit. The pooled libraries were sequenced for 250 × 250-bp cycles. Adapter trimming with default parameters was performed automatically after base calling on board the MiSeq instrument, and the resulting raw FASTQ files were uploaded to the Galaxy server (https://usegalaxy.org/) and assembled using Unicycler version 0.4.8.0 (7, 8), with the minimum contig length set to 200 bp. Each assembled genome was downloaded in a FASTA format and uploaded to the Type Strain Genome Server TYGS server (https://tygs.dsmz.de) for taxonomic assignment (9). All assemblies were submitted as whole-genome shotgun (WGS) sequencing projects to GenBank for annotation of open reading frames (ORFs), tRNAs, and rRNAs using the NCBI Prokaryotic Genome Assembly Pipeline (10). The annotation details for each isolate are presented in Table 1. Default parameters were used for all software unless otherwise noted.

**TABLE 1:** Sequencing and annotation results for the 10 endophytic and epiphytic bacteria isolated from *Lolium arundinaceum*

Organism	Genome assembly GenBank accession no.	SRA accession no.	Isolation medium^*a*^	Assembly size (bp)	Total bp sequenced	No. of contigs	Estimated coverage (×)	*N*_50_ (bp)	Assembly GC content (%)	No. of ORFs	No. of tRNAs	No. of rRNAs
Mammaliicoccus vitulinus RIT 801	JAFEMY000000000	SRR14085697	YED	2,653,076	5.09e-08	52	384	245,121	32.65	2,564	54	5
Unclassified *Micrococcaceae* RIT 802	JAFEMX000000000	SRR14085696	PD	3,866,327	4.58e-08	38	237	201,579	68.2	3,582	49	2
Unclassified *Brevibacterium* RIT 803	JAFEMW000000000	SRR14085695	LA	4,480,578	4.91e-08	13	219	696,064	62.4	3,986	49	3
Unclassified Desemzia RIT 804	JAFEMV000000000	SRR14085694	LA	3,034,747	5.25e-08	26	346	595,312	36.44	2,851	57	4
Citrobacter portucalensis RIT 805	JAFEMU000000000	SRR14085693	LA	4,768,613	4.01e-08	38	168	324,808	52.04	4,433	72	4
Staphylococcus xylosus RIT 806	JAFEMT000000000	SRR14085692	TSA	2,706,653	4.47e-08	29	330	439,484	32.58	2,548	54	6
Priestiamegaterium RIT 807	JAFEMS000000000	SRR14085691	TSA	5,749,463	3.89e-08	113	135	231,582	37.63	5,853	86	5
Unclassified *Bacillus* RIT 809	JAFEMR000000000	SRR14085690	YED	5,271,175	4.14e-08	92	157	177,226	35.34	5,431	77	8
Unclassified *Enterobacteriaceae* RIT 814	JAFEMQ000000000	SRR14085689	LA	4,602,022	4.96e-08	25	215	470,731	54.86	4,342	78	4
Leclercia adecarboxylata RIT 815	JAFEMP000000000	SRR14085688	R2A	4,734,054	4.31e-08	63	182	309,999	55.75	4,404	76	4

a YED, yeast extract-dextrose; PD, potato dextrose; LA, Luria agar; TSA, tryptic soy agar; R2A, Reasoner’s 2A agar.

### Data availability.

The whole-genome assembly, Sequence Read Archive (SRA), and annotation details for the bacterial genomes are presented in Table 1.
